# Protocol for evaluation of effects of a psychoeducational trauma-informed intervention directed at schools

**DOI:** 10.1080/20008066.2023.2263322

**Published:** 2023-10-12

**Authors:** Hussein Hamad, Amanda Angelöw, Elia Psouni

**Affiliations:** Department of Psychology, Lund University, Lund, Sweden

**Keywords:** Adverse childhood experiences, trauma informed care, intersectionality, psychoeducation, school setting, Experiencias adversas en la infancia, atención informada sobre trauma, interseccionalidad, psicoeducación, entorno escolar, 不良童年经历, 创伤知情护理, 交叉性, 心理教育, 学校环境

## Abstract

**Background:** Adverse childhood experiences (ACE) can have negative effects on cognitive, social and emotion regulation abilities, which can threaten the child’s school integration and capacity to learn. While steady relations to sensitive, understanding adults may moderate these negative outcomes, the difficulties of children with ACEs pose a major challenge for teachers, whose insufficient preparation may lead to career attrition.

**Objective:** Psychoeducational trauma-informed care (TIC) interventions targeting teachers may strengthen teacher preparation and buffer the deleterious outcomes of ACEs, yet the evidence-base for these interventions is limited. Importantly, while minority groups are overrepresented among those with ACEs and additionally risk exposure to ethno-racial trauma, TIC interventions lack a social disadvantage/discrimination perspective. The Present trial addresses these issues.

**Method:** The study protocol employs a quasi-experimental design for assessing effects of a psychoeducational TIC intervention carried out in Swedish schools by Save the Children, Sweden (SCS). We compare, for the first time, an intervention group (*N* = 160) and a control group (*N* = 160) over time (pre-intervention, immediately after, 6 and 12 months post-intervention), assessing teacher stress, compassion fatigue, self-efficacy and trauma-informed knowledge. We monitor teacher attitudes and attributions of students’ academic weaknesses and behavioural and mental difficulties. The trial is preregistered (DOI:10.17605/OSF.IO/V7SH8).

**Results:** We hope that the mitigating effects of the SCS-TIC school intervention may be independent of social category, and that the trial will additionally generate knowledge of how providers and recipients of TIC may respond to it differently depending on their social and cultural identities. As school-based TIC practices and interventions are expansively relied on as means of preventing teacher burnout and career attrition, and buffering negative consequences of ACEs for children, establishing their effects with methodological robustness is important and timely.

**Conclusion:** Such knowledge may be used to tailor and target interventions to specific populations, while ensuring maximum effectiveness.

## HIGHLIGHTS

- Psychoeducational trauma-informed care interventions targeting teachers may strengthen teacher preparation and buffer the negative outcomes of adverse childhood experiences.
- The evidence based for these interventions is insufficient, while potential interactions with the teachers’ and children’s social identities are unexplored.
- Our quasi-experimental, longitudinal, preregistered trial will address these knowledge gaps and increase the knowledge for targeted intervention.

## Abstract (es)

**Antecedentes:** Las experiencias adversas en la infancia (ACE, por sus siglas en inglés) pueden tener efectos negativos sobre las capacidades cognitivas, sociales y de regulación emocional, lo que puede amenazar la integración escolar y la capacidad de aprendizaje del niño/a. Si bien las relaciones estables con adultos sensibles y comprensivos pueden moderar estos resultados negativos, las dificultades de los niños con ACE plantean un desafío importante para los docentes, cuya preparación insuficiente puede conducir al abandono de su carrera.

**Objetivo:** Las intervenciones psicoeducativas de atención informada sobre el trauma (TIC, por sus siglas en inglés) dirigidas a los docentes pueden fortalecer la preparación docente y amortiguar los resultados nocivos de las ACE, aunque la base de evidencia para estas intervenciones es limitada. Es importante destacar que, si bien los grupos minoritarios están sobrerrepresentados entre aquellos con ACE y además corren el riesgo de estar expuestos a traumas étnico-raciales, las intervenciones de TIC carecen de una perspectiva de desventaja/discriminación social. El presente ensayo aborda estas cuestiones.

**Método:** El protocolo del estudio emplea un diseño cuasiexperimental para evaluar los efectos de una intervención psicoeducativa con TIC realizada en escuelas suecas por Save the Children, Suecia (SCS). Comparamos, por primera vez, un grupo de intervención (*N* = 160) y un grupo de control (*N* = 160) a lo largo del tiempo (pre – intervención, inmediatamente después, 6 y 12 meses después de la intervención), evaluando el estrés de los docentes y la fatiga por compasión, autoeficacia y conocimiento informado sobre el trauma. Monitoreamos las actitudes de los maestros y las atribuciones de las debilidades académicas y las dificultades mentales y de comportamiento de los estudiantes. El ensayo está prerregistrado (DOI:10.17605/OSF.IO/V7SH8).

**Resultados:** Esperamos que los efectos mitigantes de la intervención escolar SCS-TIC puedan ser independientes de la categoría social, y que el ensayo genere adicionalmente conocimiento sobre cómo los proveedores y receptores de TIC pueden responder de manera diferente dependiendo de sus identidades sociales y culturales. Dado que las prácticas e intervenciones de TIC en las escuelas son ampliamente utilizadas como medio para prevenir el agotamiento docente y el desgaste profesional, y amortiguar las consecuencias negativas de las ACE para los niños, es importante y oportuno establecer sus efectos con solidez metodológica.

**Conclusión:** Dicho conocimiento puede utilizarse para adaptar y dirigir intervenciones a poblaciones específicas, garantizando al mismo tiempo la máxima eficacia.

## Abstract (zh)

**背景：**不良童年经历（ACE）会对认知、社交和情绪调节能力产生负面影响，从而威胁到孩子的学校融入和学习能力。虽然与敏感、善解人意的成年人保持稳定的关系可能会缓和这些负面结果，但患有 ACE 的儿童的困难对教师构成了重大挑战，他们的准备不足可能会导致职业损耗。

**目的：**针对教师的心理教育创伤知情护理 (TIC) 干预措施可能可以加强教师准备并缓冲 ACE 的有害后果，但这些干预措施的证据基础有限。重要的是，虽然少数群体在 ACE 人群中所占比例过高，并且还存在遭受民族种族创伤的风险，但 TIC 干预措施缺乏社会劣势/歧视视角。本试验致力于这些问题。

**方法：**该研究方案采用准实验设计来评估瑞典救助儿童会 (SCS) 在瑞典学校进行的心理教育 TIC 干预的效果。我们首次比较干预组 (*N* = 160) 和对照组 (*N* = 160) 随着时间的推移（干预前、干预后立即、干预后 6 个月和 12 个月），评估教师压力、共情疲劳 、自我效能和创伤知识。我们监控教师的态度以及对学生学业弱点以及行为和心理困难的归因。该试验已预注册 (DOI:10.17605/OSF.IO/V7SH8)。

**结果：**我们希望 SCS-TIC 学校干预的缓解效果可能独立于社会类别，并且该试验还将进一步了解 TIC 的提供者和接受者如何根据其社会和文化身份对其做出不同的反应。由于基于学校的 TIC 实践和干预措施被广泛依赖作为防止教师倦怠和职业损耗以及缓冲 ACE 对儿童的负面影响的手段，用稳健的方法确定其效果非常重要且及时。

**结论：**这些知识可用于针对特定人群量身定制的干预措施，同时确保最大的有效性。

## 1. Background

Adversities during childhood have profound and far-reaching consequences for the child’s development and well-being, constituting a significant risk of mental illness, unemployment, poverty, and criminality in adulthood (Bellis et al., 2019; Davidson et al., 2010). Among the prevalent forms of adversity focused on in the seminal Adverse Childhood Experiences (ACEs) study (Felitti et al., 1998) were child abuse (physical, emotional and sexual), child neglect (physical and emotional), and dysfunctional parental caregiving (domestic violence, substance abuse, mental illness, criminal activity or parental absence). Other common forms of childhood adversity include loss of loved ones, poverty, peer rejection, dramatic and frequent relocations, bullying, racism, and discrimination (Bernard et al., 2021). Notwithstanding individual differences, it is not unusual that ACEs cause traumatic stress reactions with lasting detrimental effects on overall functioning (Felitti et al., 1998; Oral et al., 2015). Thus, ACEs can be considered as potentially traumatic, but whether they result in trauma or not depends on several interacting factors (Bartlett & Sacks, 2019).

Crucially, ACEs have a negative impact on cognitive and social abilities, such as attention, executive functioning, decision making, self-regulation, social competence, and communication skills, all of which are decisive for the child’s academic engagement and development (Shonkoff et al., 2012; Steiner et al., 2016; Stokes, 2022; Van der Feltz-Cornelis et al., 2019). As research has demonstrated, it is 2.5 times more likely for individuals who report four or more ACEs to suffer impairments crucial for their education (e.g. difficulties in self-regulating abilities and high levels of perceived stress) (Anda et al., 2006), leading to low achievement, absence problems, risky behaviours, suspension, and early drop out or expulsion (Bellis et al., 2018; Cook et al., 2005; Delaney-Black et al., 2002; Ford et al., 2006; Porche et al., 2016). However, research demonstrates that caring, attentive parents and other important adults, such as teachers, can play a crucial role in protecting children from the deleterious consequences of early childhood adversities, serving to mitigate the risks on further development (Van der Kolk, 2005; Verschueren & Koomen, 2012).

In fact, quality in the child – teacher relationship at preschool predicts academic and behavioural outcomes at school age (Broberg et al., 2012). Conversely, in the absence of attentive and supportive significant adults, ACEs may lead children to perceive adults as potential threats, impeding their ability to form healthy relationships, and students with ACEs are more prone to encountering conflicts with school staff and teachers (Fazel et al., 2014). The ecology of human development model (Bronfenbrenner & Morris, 2006) highlights this intricate and interconnected interplay between multiple contexts in the child’s growing environment and describes how alterations in any one system might influence child development in several ways. Thus, children with ACEs are in the forefront of risk for strained and conflictual relationships to teachers and other adults in the school setting, which may exacerbate and amplify the negative outcomes of their ACEs.

Difficult student behaviour presents a significant challenge for teachers, evidenced by the substantial number of teachers who cite the difficulty of handling students with behavioural challenges as the primary reason for leaving the profession within four years (Greene, 2008). Thus, many teachers may feel unprepared when faced with challenging child behaviours. Importantly, teachers who themselves have ACEs are at risk of re-traumatisation, as interactions with traumatised children may bring their own traumatic memories and difficult emotions forward, and lead to burnout (Fazel et al., 2014). Indeed, there is evidence that a quarter of new teachers will leave the profession within one year, possibly highlighting the toxic stress from unpreparedness in handling increasing numbers of children with difficulties, and, for some, simultaneous secondary traumatisation (Aloe et al., 2014). Perceptions of being poorly equipped to cope with student challenging and disruptive classroom behaviours, and lack of access to support systems within the school, is another highlighted reason for this pattern of career attrition among teachers (Fazel et al., 2014). Research suggests that conflictual teacher-student relationships might in part stem from miscommunication rooted in limited understanding of the impact of ACEs on learning abilities (Di Lemma et al., 2019). Thus, the urgent need of addressing teachers’ knowledge and understanding of how ACEs and trauma affect children in the educational environment is pivotal in efforts to limit the consequences of trauma.

### 1.1. Psychoeducational trauma-informed care intervention in the school setting

Since having at least one significant relationship characterised by trust and security increases child resilience (Masten, 2014), interventions aimed at the teacher–child relationship may buffer behavioural problems and poorer school performance later on, potentially contributing to the children’s long-term resilience. Psychoeducational trauma-informed care (TIC) interventions directed to teachers and other school personnel is such support. While referred to as trauma-informed care, TIC interventions encompass the entire range of challenges associated with ACEs and trauma. TIC interventions vary widely but the overall aim is to promote safety and resilience in children who have been exposed to adversities, whereas the psychoeducational TIC interventions directed at caregivers focus on increasing caregivers’ recognition, knowledge, and awareness of childhood trauma (Middleton et al., 2019; SAMHSA, 2014).

Psychoeducational TIC interventions directed at teachers and other school personnel are increasingly used internationally (Avery et al., 2021), and studies indicate decreased levels of secondary traumatic stress and burnout (Sprang & Garcia, 2022), increased trauma-informed attitudes among school personnel, including among teachers with positive trauma-informed attitudes pre-training (MacLochlainn et al., 2022), and a strengthened proactive and supportive classroom environment (Stokes, 2022) post-intervention. One TIC intervention directed at teachers and school personnel is based on the *Three pillars of transforming care* model (Bath, 2015). By providing teachers with knowledge about trauma, the programme hopes to help them understand and meet the needs of children and adolescents who have experienced trauma (Bath & Seita, 2019). TIC programmes based on the Three pillars model in out-of-home care result in higher levels of caregiver efficacy and decreased child behavioural difficulties (Lotty et al., 2020; Purvis et al., 2015). However, besides an unpublished pilot with a pedagogical perspective (Herbert et al., 2017), the Three pillars interventions in the school setting have not been evaluated.

### 1.2. Social discrimination and childhood adversity

While there is appreciation of, and promise in, TIC interventions, a notable limitation stems from insufficient consideration of how social categories and social group memberships interact and shape children’s experiences of ACEs, including collective experiences of trauma (Ginwright, 2018; Middleton et al., 2019). Intersectionality, a term coined by critical race theorist Kimberlé Crenshaw in the late 80’s to capture the unique experience of violence against black women, highlights the compounding effects in how social identities intersect and shape our unique experiences. Crenshaw (1989) argued that violence against black women could not be fully grasped if examined through the lens of either racism or sexism, but rather through considering racism and sexism conjointly.

Arguably, then, the comprehensive understanding and effective action for buffering long-term effects of ACEs on children requires consideration of the intricate intersections of social identities and segments of socio-economic strata. These intersections shape and govern our daily lives, and influence both our unique experiences of adversity and those of resilience. For instance, research suggests that racism is so ubiquitous in the lives of black youth that, independent of the presence or absence of other ACEs, they encounter an average of five instances of racial discrimination per day (English et al., 2020), constituting a major source of stress in the lives of members of ethnic and racial minority groups (Bernard et al., 2021). Consistently, research has emphasised that the profound and compounding deleterious effects of everyday experiences of racial discrimination may be best understood through the perspective of trauma and traumatic stress (Saleem et al., 2020). Incorporating knowledge about racism, discrimination and minority group-related experiences within an extended, culturally-informed framework of ACEs and TIC is thus essential (Bernard et al., 2021).

### 1.3. Childhood adversity and school-based trauma-informed care intervention in Sweden

Experiences of adversities in childhood are common also in a Swedish context. Among Swedish youth, 15% reported experiences of some form of negative exposure in childhood, and 10% reported two or more types of negative exposure before the age of 12 (Hagborg et al., 2018). In response to the Swedish government’s assignment (U2019/03787/S) to support the quality of education for newly arrived, non-Swedish-speaking children, and acknowledging the need for enhanced trauma knowledge among educational staff (Skolverket, 2023), Save the Children Sweden (SCS) was commissioned by the Swedish National Agency of Education in 2017 to train school personnel in a psychoeducational TIC school programme based on the Three Pillars intervention. Notably, among the conditions of the Swedish National Agency of Education for enrolling schools to the programme are that the school (a) has newly arrived children and children with immigrant background, and (b) is located in a socially vulnerable area.

Evidently, experiences of racism and racial discrimination are relevant in the Swedish setting. Statistics Sweden reports substantial disparities between children with a foreign background and those with a native Swedish background across various crucial aspects of life, including socio-economic status, family dynamics and educational opportunities (SCB, 2020). For example, more than 40% of children (0–17 years) with foreign (i.e. immigrant) background live in areas burdened by socio-economic challenges, compared to 5% of children with Swedish background. A recent report by SCS (Rosenlundh et al., 2021) indicates that every second child has witnessed racism in the school setting, and every fourth child reported to have experienced discrimination and/or harassment. A fifth of all children reported being afraid of being bullied because of their ethnic background. Notably, the children perceived adults as passive, seldom intervening or doing anything to stop racism in school (Rosenlundh et al., 2021). The school environment appears to be a primary setting where racism against children is perpetrated (Rosenlundh et al., 2021). Thus, an ethnic and culturally informed ACEs framework is highly warranted.

### 1.4. Aims and objectives of the planned study

Despite paucity of empirical evidence of the effectiveness of TIC interventions, clinical research data indicate that an increased knowledge among caregivers concerning trauma and TIC practices leads to changed TIC attitudes (Brown et al., 2012). A recent meta-analysis of TIC interventions within the child welfare system indicated that caregivers reported reduced child PTSD symptoms and behavioural problems but only moderate improvement on children’s wellbeing (Zhang et al., 2021). However, while recent international studies regarding trauma-focused interventions are encouraging (Angelöw et al., 2023; Izzo et al., 2016; Purvis et al., 2015), most evaluations of TIC interventions suffer from methodological shortcomings and lack robust methods such as a control group or longer-term follow ups (Fondren et al., 2020; Hanson & Lang, 2016). In a school setting, the Three pillars intervention directed at teachers (Bath et al., 2021) has never been evaluated.

Therefore, the purpose of this study is to assess the effect of psychoeducational interventions in trauma-informed care directed at teachers. Building on the limited existing empirical evidence (MacLochlainn et al., 2022; Zhang et al., 2021) we expect that, after training in TIC, participants will report changed attitudes toward trauma-informed care and related practices (H1). These effects will be associated with increased perceived teacher efficacy, classroom management strategies (H2). Moreover, we expect decreased reported burnout and compassion fatigue symptoms (H3). Finally, we expect that these effects will be present also 6-months post-training (H4). Due to the underexplored nature of psychoeducational TIC interventions in terms of interactions with awareness about ethno-racial trauma and community specific experiences of ACEs, we refrain from making predictions concerning how the intervention might influence awareness about such socioeconomic and intersectional aspects of ACEs and trauma.

## 2. Methods and design

### 2.1. The trauma informed care (TIC) psychoeducational intervention for schools

The SCS psychoeducational TIC school programme (Bath et al., 2021) provides a pedagogical framework for understanding how significant adults create conditions for healing and recovery in children who are exposed to trauma. With an aim of developing teachers’ ability to respond to pupils with a trauma-informed approach, the 5-day programme builds around the themes of safety, connections, and coping (Bath, 2015), and provides education about compassion fatigue and secondary traumatic stress, practical TIC training, and workshops on TIC implementation at an organisational level. TIC-ambassadors continue to work closely with the principal and support the TIC organisational implementation after finished training. For participation in the SCS-TIC programme, school principals must apply to the Swedish National Agency for Education, who then, based on the school’s written application and interviews with the school principal, select (prioritise) based on parameters including implementation capacity and the likely prevalence of trauma in the pupil population.

### 2.2. Study design

We employ a quasi-experimental design with four measurements: Pre-training baseline (T1), directly after termination of training (T2), six months (T3) and twelve months (T4) after baseline. An experimental group (training in SCS TIC-programme) will be compared to a control group (no training in SCS TIC-programme). Participants in the control group will receive the same questionnaires with the same time interval between the measurement occasions.

### 2.3. Participants and recruitment

Participants will be teachers and other professionals in the school context (principals, special teachers, school nurses) working in Swedish schools, from primary school to high school. Participants in the experimental group (*N* = 160) will be recruited from schools who participate in the SCS-TIC training programme. Sixteen (16) training groups consisting of 35 people each participate in the SCS-TIC training programme each semester. Participants will be excluded if they do not complete the programme. An equally large control group (*N* = 160) will be recruited from schools not receiving the SCS-TIC programme. For recruitment, participants in the experimental group will be contacted through collaboration with SCS. Participants in the control group will be invited to the study through e-mail and targeted on social media platforms.

### 2.4. Data collection

For data collection, we use a digital platform that sends invitations to participation and unique personalised links via email/SMS, allowing participants to respond in their own time, via smartphone, tablet or computer. Data is safely and reliably combined by the digital platform software, without personal codes or human handling, thereby maximising confidentiality and minimising risk of error. It will be possible for participants to listen to the survey questions and information about the study.

Given that the experimental group is determined by the standardised procedure of the Swedish National Agency for Education who handles the applications for participation in the SCS-TIC programme, it cannot be claimed that the experimental group is randomly selected. Nevertheless, although access to school (socioeconomic and ethnic) data is restricted by current state regulations, we strive to match the schools that contribute to the experimental group with schools of similar student population composition (class sizes, proportion of immigrant students), geographic location (i.e. urban or suburban-located schools), and socioeconomic status (i.e. location of the school) in the control group. Schools in the control group are randomly selected among those who match on the above criteria, based on a draw.

### 2.5. Primary outcome measures

Levels of stress and compassion fatigue are measured by the Professional Quality of Life scale (PROQOL 5: Stamm, 2009), which examines the potential impact from helping others who have experienced trauma and adversities. The scale consists of 30 items divided into three subscales (Compassion satisfaction, *α* = .88, Burnout, *α* = .75 and Compassion fatigue, *α* = .81), with high internal consistency (Stamm, 2010).

Teacher self-efficacy in the classroom environment is measured by the Teachers’ Sense of Efficacy Scale (TSES: Klassen et al., 2014), addressing the distinct domains of pupil engagement, instructional practices and classroom management, with high internal consistency (*α* = .86; Klassen et al., 2014).

The Knowledge and Beliefs’ Survey (KBS: Murray, 2014) assesses self-efficacy, tolerance of child misbehaviour and knowledge of trauma-informed care and consists of 33 items and three subscales (Trauma-Informed Parenting, Tolerance of Misbehaviour, and Parenting Efficacy), all with satisfactory internal consistency (Cronbach’s a .75–.83; Murray, 2014; Sullivan et al., 2016). KBS is commonly used for assessment in the foster care setting. Here, we use a subset of 17 items, adapted to apply in the school setting (e.g. *I routinely think about how students with traumatic experiences are physically safe at our school, but might not feel safe*). The 16 items that specifically concerned out-of-home care (e.g. *I feel confident talking with my child about his/her feelings about his/her biological parents*) were removed.

Awareness about intersectional disparities concerning how ACEs affect individuals and the potential traumatic experiences of racism and discrimination is operationalised utilising three different constructs:

- Beliefs and attributions of low academic achievement among students with a non-Swedish background are assessed by the Teacher Attributions of Student Academic Achievement Scale, which encompasses 8 items. The scale was developed and validated in a prior school study (Mohammadi & Wolgast, in preparation) and there is evidence of high internal consistency.
- Beliefs and attributions concerning mental illness among students with a non-Swedish background are assessed by the Teacher Attributions of Student Mental Health Scale, developed for the purpose of the present project. The scale comprises 16 items organised in two subscales: internal (i.e. individual) attributions and external (i.e. situational) attributions related to the higher risk of mental health problems among non-Swedish youth, compared to their Swedish counterparts. The subscales demonstrate high internal consistency (Hamad et al., in preparation).
- Negative attitudes toward immigrants are measured by the modern Racism Scale, a 9-item measure that assesses covert forms of racist attitudes toward immigrants (as opposed to blatant and traditional forms of racism). The scale was adapted for the Swedish context with high internal consistency (Cronbach’s *α* = .82; Akrami et al., 2000).

### 2.6. Background variables

Background variables will include participants’ age, mother language, gender, profession, work experience, ethnicity, and social class.

### 2.7. Power/sample size calculations, data analysis plan and attrition analyses

A power analysis employing R and G*Power with an aimed .80 power, significance level of .05 and an effect size of *d* = .5, yielded a sufficient sample size of *N* = 99 for our basic analyses. Estimating a drop-rate of about 40% over time, perhaps even higher for the control group, we determined a target sample size of *N* = 160 for the experimental group and *N* = 160 for the control group.

Data from all measures will be analysed using R (R Core Team, 2021) and JAMOVI (The Jamovi Project, 2022). Attrition analyses will be conducted, and missing data among participants who have completed all four measurements will be handled either utilising multiple imputation or by applying maximum likelihood estimation when employing the mixed linear model analysis. Independent *t*-tests will assess potential differences between experiment and control group concerning outcome measurements (e.g. perceived teacher efficacy, classroom management strategies, burnout and compassion fatigue symptoms) at baseline. For hypothesis testing, we will employ mixed linear model analyses of repeated measures to estimate fixed (i.e. the trauma-informed care training) and random (e.g. participants’ social class, gender, ethnicity) effects of training on the study outcome variables, comparing both within individuals (repeated measures analysis) and between groups (experimental versus control group measures at each timepoint). Interaction factors in the models are used for estimating potential moderation by ethnicity, socioeconomic status and other characteristics. For example, a three-way interaction of gender, ethnicity and social-class may uncover intersectional outcomes particularly for women with non-Swedish backgrounds that might indicate a heightened trauma-informed care approach to vulnerable students independently of whether they are in the experimental or control group.

## 3. Discussion

Sparked by the extensive empirical demonstration of pervasive and detrimental consequences of ACEs across the lifespan, increased interest in TIC practices and interventions is evident across various service sectors (Kenny et al., 2017). Children burdened with ACEs often face difficulties resulting from dismissive attitudes, incorrect assumptions and erroneous attributions made by important adults in their proximity, which could further exacerbate this intricate and complex interplay of a trauma induced cycle of negative outcomes (Felitti et al., 1998). In the school context, teachers and school staff experience stress and burnout stemming from encounters with students’ disruptive and challenging behaviours (Fazel et al., 2014), suggesting limitations in preparedness to support students troubled by ACEs.

Intervention to enhance trauma-informed care and practices at school is thus especially warranted (Masten, 2014). As Van der Kolk (2014) stated, a sense of safety among people is perhaps the most crucial contribution to mental health, and social support is most likely the most powerful protection against the toxic consequences of stress and trauma. Successful intervention will not only mitigate the suffering of affected individual children but also reduce expensive dispenses for society. However, while TIC practices and interventions may have promising potential to make a difference (MacLochlainn et al., 2022; Zhang et al., 2021), effects of TIC interventions in a school context are still not empirically evidenced, and the present study will assess effects in ways that hopefully guarantee methodological robustness and fill an important gap in the trauma literature.

Crucially, although empirical work has demonstrated how early adversities might affect individuals differently, emphasising the risk of additional burden from social inequalities and discrimination and proposing models of healing from ethno-racial trauma (Bernard et al., 2021; Chavez-Dueñas et al., 2019), the conventional TIC-framework is lacking behind in incorporating these perspectives (Saleem et al., 2020). Notably, studies that employ expanded ACE frameworks, that encompass potentially traumatic events relevant to experiences among ethnic and racially diverse minorities, demonstrate that these frameworks are more endorsed by members of minority groups compared to non-minority groups (Cronholm et al., 2015), and result in more accurate reports of ACEs from children and youth from minority groups (Maguire-Jack et al., 2020). Thus, understanding how social inequalities and discrimination may interact with the outcomes of TIC intervention is essential, as interventions may be futile for children in critical need of receiving adequate support if their calls for help are not recognised as expressions of trauma.

Despite the trial’s rigorous design, we acknowledge certain methodological limitations. The absence of intervention for the control group is a weakness. Additionally, participants may report positive trauma-informed attitudes as a result of self-fulfilling prophecies to enhance the effects of training. As schools and school teachers invest substantial resources in terms of time, energy, commitment in TIC, and related work structures in the school, another potential source of bias is from ‘escalation of commitment’ denoting the tendency to declare continued commitment in endeavours invested in. Importantly, the trial focuses on the perspective of the teachers as providers of TIC. Research has demonstrated that teachers may not always ‘practice what they preach’, and reported beliefs, attitudes and behaviours may thus not be aligned with the lived experiences of the students (Mansour, 2013; Tobin et al., 1994). Lastly, given the trial longitudinal design, participant attrition may pose a threat to the data.

Since the decision of which schools will be given TIC-training is preset by the Swedish government, it is not possible to guarantee full randomisation.

### 3.1. Ethical considerations

The Swedish Ethical Review Authority has reviewed and endorsed the present study (Dnr 2022-03446). Ethical and professional guidelines are followed at all times. We provide presumptive participants with comprehensive information, prior to enrolment, concerning data procedures, about the data that will be collected about them and how it will be handled, and about the option to withdraw at any point. The study places strong emphasis on ensuring full confidentiality, promptly anonymizing data, and handling participant lists safely. Sensitive personal data will not be collected.

None of the proposed measurements are known to cause harm. Negative thoughts or feelings that may arise during the psychoeducation are likely to be short-lasting. Moreover, the educators inform of the possibility to take a break or leave the room prior to exposure to material that may be interpreted as disturbing. In fact, drawing from our previous experiences, engaging in the intervention may have positive effects such as providing insights and thoughts that might evoke efficacy in professional practice. Nonetheless, to ensure additional support and aftercare, a clinical psychologist outside of the study will be accessible by phone or email.

## 4. Conclusion: added value

The present trial is likely to make a valuable contribution to the literature of trauma-informed care, using a quasi-experimental design with several repeated measures and comparisons with a control group. We hope that the effects of TIC intervention at schools are independent of social category, and that our trial will additionally generate knowledge of how providers and recipients of TIC may respond differently depending on their social and cultural identities, such that may be used to tailor and target interventions to specific populations, while ensuring maximum effectiveness.

## Author statement

*Hussein Hamad:* Conceptualisation; methodology; writing – original draft preparation; writing – review and editing.

*Amanda Angelöw:* Conceptualisation; methodology; writing – original draft preparation; writing – review and editing.

*Elia Psouni:* Conceptualisation; methodology; writing – original draft preparation; writing – review and editing; supervision; project administration; funding acquisition.

The authors have read and agreed to the published version of the manuscript.

## Data Availability

The trial is preregistered (doi:10.17605/OSF.IO/V7SH8).

## Open Scholarship

This article has earned the Center for Open Science badges for Open Data, Open Materials and Preregistered. The data and materials are openly accessible at https://osf.io/c72b6/, https://osf.io/c72b6/ and https://osf.io/c72b6/registrations.
